# The C-terminal sequences of Bcl-2 family proteins mediate interactions that regulate cell death

**DOI:** 10.1042/BCJ20210352

**Published:** 2024-07-10

**Authors:** Dang Nguyen, Elizabeth Osterlund, Justin Kale, David W. Andrews

**Affiliations:** 1 Department of Medical Biophysics, Faculty of Medicine, University of Toronto, Toronto, Canada; 2 Biological Sciences Platform, Odette Cancer Program, Sunnybrook Research Institute, Toronto, Canada; 3 Department of Biochemistry and Biomedical Sciences, Faculty of Health Science, McMaster University, Hamilton, Canada; 4 Department of Biochemistry, Faculty of Medicine, University of Toronto, Toronto, Canada

**Keywords:** apoptosis, Bax, Bcl-2, BH3-only proteins, protein–protein interactions, transmembrane domain

## Abstract

Programmed cell death via the both intrinsic and extrinsic pathways is regulated by interactions of the Bcl-2 family protein members that determine whether the cell commits to apoptosis via mitochondrial outer membrane permeabilization (MOMP). Recently the conserved C-terminal sequences (CTSs) that mediate localization of Bcl-2 family proteins to intracellular membranes, have been shown to have additional protein-protein binding functions that contribute to the functions of these proteins in regulating MOMP. Here we review the pivotal role of CTSs in Bcl-2 family interactions including: (1) homotypic interactions between the pro-apoptotic executioner proteins that cause MOMP, (2) heterotypic interactions between pro-apoptotic and anti-apoptotic proteins that prevent MOMP, and (3) heterotypic interactions between the pro-apoptotic executioner proteins and the pro-apoptotic direct activator proteins that promote MOMP.

## Introduction

When a eukaryotic cell is faced with overwhelming cellular stress that surpasses the capacity to repair and recover, the mitochondrial pathway of apoptosis is triggered [1,2]. This crucial decision is regulated by the Bcl-2 family of proteins, which serve as the master switch at the mitochondrial outer membrane (MOM) [3–5]. Once the switch is flipped, the MOM becomes permeabilized by one or more of the executioner proteins from the Bcl-2 family (Bax, Bak, and Bok), thereby committing the cell to apoptosis. These globular multi-domain pore-forming proteins are synthesized in inactive monomeric forms that upon activation oligomerize within the MOM to form large complexes that permeabilize the lipid bilayer. This event results in the release of pro-apoptotic factors, from the mitochondrial intermembrane space into the cytosol, thereby activating cysteine aspartyl proteases (caspases) leading to a caspase activation cascade that results in the proteolytic degradation of cellular components, culminating in cell death [6–8]. Most Bcl-2 family members possess a C-terminal sequence (CTS) that localizes and binds the protein to intracellular membranes such as the MOM and endoplasmic reticulum (ER) [9–12]. Beyond localization, the precise roles of these CTSs are not well understood [13], and recent evidence suggests that several of these sequences have unanticipated functions [14–18]. Here we review our current understanding of the CTSs in Bcl-2 family members, highlighting the potential importance of recently discovered functions of these sequences and how they govern the interactions among Bcl-2 family members.

Generally considered as the point of no return in committing the cells to apoptosis, MOM permeabilization (MOMP) involves complex interactions amongst a network of Bcl-2 family proteins, resembling an intricate ‘dance’ [19]. Critically, the specific interactions that occur in any one cell are determined by the abundance, localization and relative affinities of the various Bcl-2 family proteins. The relative affinities for such interactions or ‘dances’ are mediated by common sequences and domains acting as receptors and ligands, shared between Bcl-2 proteins. The 4 different Bcl-2 homology (BH) motifs (BH1, BH2, BH3, and BH4) form the bulk of these binding sites for Bcl-2 proteins. Bcl-2 members with all four BH-motifs are referred to as multi-BH motif (or multi-BH domain) proteins and include both anti-apoptotic and executioner proteins. Of the four BH motifs, the BH3 motif is the only one that is present in all Bcl-2 family members and BH3 deletion abolishes the apoptotic regulatory function of most Bcl-2 members. In multi-BH motif proteins, the BH1, BH2, and BH3 motifs are organized into a hydrophobic region called the ‘canonical BH3-binding groove’ that acts as a receptor that binds to a BH3 motif (the ligand) of binding partners. In general, direct interactions between family members either facilitate or hinder the oligomerization of pore forming multi-BH motif executioner proteins in the MOM to promote or inhibit MOMP respectively [20]. In the direct activation model, to induce MOMP, specific pro-apoptotic BH3-only proteins, so called because they only have the BH3 motif, use the BH3 motif to directly bind to BH3-binding sites on Bax or Bak. Binding causes conformational changes in Bax/Bak that result in them forming dimers that subsequently link up to create membrane-permeabilizing structures (arcs and rings) in the MOM [21–23]. The activator BH3-only proteins, including Bim, Bid and Puma, also bind to the canonical BH3-binding grooves of the anti-apoptotic multi-BH motif proteins Bcl-2, Bcl-XL, Bcl-W, Mcl-1, and Bfl-1/A1, resulting in inhibition of both proteins via mutual sequestration [14,16]. Importantly, the CTSs of Bim and Puma not only localize these proteins to intracellular membranes to facilitate binding the pro-apoptotic executioners and the anti-apoptotic proteins but also directly participate in these protein-protein interactions by binding to sites other than the BH3 motif [14–16]. The ‘free’ or uninhibited anti-apoptotic proteins also directly bind to and inhibit activated Bax/Bak thereby limiting dimerization/oligomerization [24,25]. Binding of anti-apoptotic proteins to the MOM via the CTSs directly contributes to how effectively Bax and Bak are inhibited [26].

Another group of BH3-only proteins, known as sensitizer proteins, including Bad, Noxa, Bmf, Hrk, and Bik, bind to and inhibit anti-apoptotic proteins thereby displacing the activated executioner proteins that are bound to them. In the indirect activation model for Bax and Bak the executioner proteins are constitutively activated or activated independently of the direct-activator BH3-only proteins, and MOMP is regulated primarily through inhibition of anti-apoptotic proteins by sensitizer and activator proteins [27,28]. Binding of sensitizer proteins can also displace direct activator BH3-only proteins from anti-apoptotic proteins. In a closely related mechanism, the CTSs of the anti-apoptotic protein Bcl-XL contribute to Bcl-XL dimerization and thereby enable allosteric regulation of Bcl-XL whereby both sensitizer and activator BH3-only proteins are bound by the Bcl-XL dimer [29]. In this scenario, binding of the sensitizer protein Bad to one Bcl-XL monomer changes the interaction of the other Bcl-XL monomer with tBid such that the bound tBid then activates Bax and Bak [29,30]. Although the role of direct versus indirect activation of Bax/Bak in cell death is still debated [32], in both models the CTSs of executioner proteins are important for MOMP as truncating the CTSs significantly reduced the cell-killing activities compared with full-length proteins [33–35]. This loss of function is generally ascribed to improper subcellular localization of the protein which alters both the available Bcl-2 family protein binding partners and the apparent affinities of the interactions between the proteins [19,35,36].

Recent evidence suggests that the CTSs also directly mediate important protein-protein interactions among Bcl-2 family members, thereby regulating mitochondrial permeability and influencing cell fate. These interactions include: (1) homotypic interactions among the CTSs of Bax, Bak, and Bok; (2) heterotypic interactions between the CTSs of Bax, Bak and Bok with the CTSs of anti-apoptotic proteins; (3) interactions between the CTSs of the BH3-only proteins Bim and Puma with anti-apoptotic proteins that confer resistance to inhibitors of anti-apoptotic proteins; and (4) interactions of BH3-only protein CTSs with Bax and Bak important for activation of these executioner proteins. Here we briefly describe the canonical function of CTSs in localizing Bcl-2 proteins to subcellular organelles and the challenges encountered when studying protein-protein interactions mediated by the CTSs due to the canonical function of CTSs in localizing Bcl-2 proteins to subcellular membranes (Table 1). We then discuss in depth the non-canonical functions of CTSs in mediating protein-protein interactions among Bcl-2 family members (Table 2).

**Table 1.** List of the C-terminal sequences (last 30 residues) from Bcl-2 and non-Bcl-2 proteins.

Protein category	Uniprot ID	Protein	C-terminal sequence																														ΔF_sm: the predicted membrane-binding free energy for a vesicle with a 50 nm radius (kJ/mol) [37]	Membrane-binding prediction [37] *Sensor = only bind to curved membranes*Binder = binds to membrane without curvature selectivity*Non-binder = no binding to membranes	Transmembrane prediction [38]	Tail-anchor prediction (membrane embedded in red) [39]	Consensus prediction on membrane binding	Experimental validation	PMID
			1	2	3	4	5	6	7	8	9	10	11	12	13	14	15	16	17	18	19	20	21	22	23	24	25	26	27	28	29	30							
Bcl-2 - BH3-only proteins	O43521	Bim (Human)	L	N	N	Y	Q	A	A	E	D	H	P	R	M	V	I	L	R	L	L	R	Y	I	V	R	L	V	W	R	M	H	−66.679	Binder	N	N	Peripheral binding	Iodine quenching of NBD-labelled CTS	30860026
							*h*	*h*	*h*					*h*	*h*	*h*	*h*	*h*	*h*	*h*	*h*	*h*	*h*	*h*	*h*	*h*	*h*	*h*	*h*	*h*									
	Q9BXH1	Puma (Human)	R	P	S	P	W	R	V	L	Y	N	L	I	M	G	L	L	P	L	P	R	G	H	R	A	P	E	M	E	P	N	−16.322	Sensor	N	N	Peripheral binding	C-terminal fusion of Venus to Puma	37078707
						*h*	*h*	*h*	*h*	*h*	*h*	*h*	*h*	*h*	*h*	*h*	*h*																						
	P55957	Bid (Human)	L	L	R	D	V	F	H	T	T	V	N	F	I	N	Q	N	L	R	T	Y	V	R	S	L	A	R	N	G	M	D	−9.833	Non-binder	N	N	Non-binding	None	N/A
				*h*	*h*	*h*	*h*	*h*	*h*	*h*	*h*	*h*	*h*	*h*	*h*	*h*	*h*	*h*	*h*	*h*	*h*	*h*	*h*	*h*	*h*	*h*	*h*	*h*	*h*										
	Q13323	Bik (Human)	V	S	C	E	Q	V	L	L	A	L	L	L	L	L	A	L	L	L	P	L	L	S	G	G	L	H	L	L	L	K	−69.173	Binder	Y	Y	Transmembrane	Resistant to sodium carbonate extraction	11884414
					*h*	*h*	*h*	*h*	*h*	*h*	*h*	*h*	*h*	*h*	*h*	*h*	*h*	*h*	*h*	*h*	*h*	*h*	*h*	*h*			*h*	*h*	*h*	*h*	*h*								
	Q92934	Bad (Human)	T	Q	M	R	Q	S	S	S	W	T	R	V	F	Q	S	W	W	D	R	N	L	G	R	G	S	S	A	P	S	Q	−31.789	Binder	N	N	Peripheral binding	None	N/A
									*h*	*h*	*h*	*h*	*h*	*h*	*h*	*h*	*h*	*h*	*h*	*h*																			
	Q13794	Noxa (Human)	C	A	T	Q	L	R	R	F	G	D	K	L	N	F	R	Q	K	L	L	N	L	I	S	K	L	F	C	S	G	T	−23.275	Sensor	N	N	Peripheral binding	None	N/A
				*h*	*h*	*h*	*h*	*h*	*h*	*h*	*h*	*h*	*h*	*h*	*h*	*h*	*h*	*h*	*h*	*h*	*h*	*h*	*h*	*h*	*h*	*h*	*h*	*h*											
	Q96LC9	Bmf (Human)	N	Q	N	R	V	W	W	Q	I	L	L	F	L	H	N	L	A	L	N	G	E	E	N	R	N	G	A	G	P	R	−11.735	Non-binder	N	N	Non-binding	None	N/A
						*h*	*h*	*h*	*h*	*h*	*h*	*h*	*h*	*h*	*h*	*h*	*h*	*h*	*h*	*h*				*h*	*h*														
	O00198	Hrk (Human)	P	G	A	L	P	T	Y	W	P	W	L	C	A	A	A	Q	V	A	A	L	A	A	W	L	L	G	R	R	N	L	−49.406	Binder	Y	Y	Transmembrane	Acrylamide quenching of Tryptophan in Hrk CTS peptide	17434443
									*h*	*h*	*h*	*h*	*h*	*h*	*h*	*h*	*h*	*h*	*h*	*h*	*h*	*h*	*h*	*h*	*h*	*h*	*h*	*h*	*h*										
	Q14457	Beclin-1 (Human)	S	E	E	Q	W	T	K	A	L	K	F	M	L	T	N	L	K	W	G	L	A	W	V	S	S	Q	F	Y	N	K	−49.608	Binder	N	N	Non-binding	None	N/A
				*h*	*h*	*h*	*h*	*h*	*h*	*h*	*h*	*h*	*h*	*h*	*h*	*h*	*h*	*h*	*h*	*h*	*h*	*h*	*h*	*h*	*h*	*h*	*h*	*h*	*h*										
Bcl-2 - executioner proteins	Q07812	Bax (Human)	S	Y	F	G	T	P	T	W	Q	T	V	T	I	F	V	A	G	V	L	T	A	S	L	T	I	W	K	K	M	G	−36.414	Binder	Y	N	Buried when Bax is activated	IASD-labeling of CTS	25228770
										*h*	*h*	*h*	*h*	*h*	*h*	*h*	*h*	*h*	*h*	*h*	*h*	*h*	*h*	*h*	*h*	*h*	*h*	*h*	*h*	*h*	*h*								
	Q16611	Bak (Human)	N	L	G	N	G	P	I	L	N	V	L	V	V	L	G	V	V	L	L	G	Q	F	V	V	R	R	F	F	K	S	−58.087	Binder	Y	Y	Transmembrane	IASD-labeling of CTS	25744027
							*h*	*h*	*h*	*h*	*h*	*h*	*h*	*h*	*h*	*h*	*h*	*h*	*h*	*h*	*h*	*h*	*h*	*h*	*h*	*h*	*h*	*h*	*h*	*h*									
	Q9UMX3	Bok (Human)	P	G	L	R	S	H	W	L	V	A	A	L	C	S	F	G	R	F	L	K	A	A	F	F	V	L	L	P	E	R	−53.299	Binder	Y	N	Transmembrane	None	N/A
								*h*	*h*	*h*	*h*	*h*	*h*	*h*	*h*	*h*	*h*	*h*	*h*	*h*	*h*	*h*	*h*	*h*	*h*	*h*	*h*	*h*											
Bcl-2 - anti-apoptotic proteins	P10415	Bcl-2 (Human)	F	D	F	S	W	L	S	L	K	T	L	L	S	L	A	L	V	G	A	C	I	T	L	G	A	Y	L	G	H	K	−34.147	Binder	Y	Y	Transmembrane	IASD-labeling of CTS	15149601
							*h*	*h*	*h*	*h*	*h*	*h*	*h*	*h*	*h*	*h*	*h*	*h*	*h*	*h*	*h*	*h*	*h*	*h*	*h*	*h*	*h*	*h*	*h*										
	Q07820	Mcl-1 (Human)	V	E	D	L	E	G	G	I	R	N	V	L	L	A	F	A	G	V	A	G	V	G	A	G	L	A	Y	L	I	R	−34.754	Binder	Y	N	Transmembrane	None	N/A
						*h*	*h*	*h*	*h*	*h*	*h*	*h*	*h*	*h*	*h*	*h*	*h*	*h*	*h*	*h*	*h*	*h*	*h*	*h*	*h*	*h*	*h*	*h*	*h*	*h*	*h*								
	Q07817	Bcl-XL (Human)	R	K	G	Q	E	R	F	N	R	W	F	L	T	G	M	T	V	A	G	V	V	L	L	G	S	L	F	S	R	K	−45.371	Binder	Y	Y	Transmembrane	IASD-labeling of CTS	24616095
						*h*	*h*	*h*	*h*	*h*	*h*	*h*	*h*	*h*	*h*	*h*	*h*	*h*	*h*	*h*	*h*	*h*	*h*	*h*	*h*	*h*	*h*	*h*	*h*	*h*									
	Q92843	Bcl-W (Human)	E	G	N	W	A	S	V	R	T	V	L	T	G	A	V	A	L	G	A	L	V	T	V	G	A	F	F	A	S	K	−28.414	Binder	Y	Y	Transmembrane	None	N/A
						*h*	*h*	*h*	*h*	*h*	*h*	*h*	*h*	*h*	*h*	*h*	*h*	*h*	*h*	*h*	*h*	*h*	*h*	*h*	*h*	*h*	*h*	*h*	*h*	*h*									
	Q16548	Bcl2-A1 (Human)	K	K	F	E	P	K	S	G	W	M	T	F	L	E	V	T	G	K	I	C	E	M	L	S	L	L	K	Q	Y	C	−23.355	Sensor	N	N	Peripheral binding	None	N/A
											*h*	*h*	*h*	*h*	*h*	*h*	*h*	*h*	*h*	*h*	*h*	*h*	*h*	*h*	*h*	*h*	*h*	*h*	*h*	*h*	*h*								
	Q9HD36	Bcl-B (Human)	P	L	A	F	W	R	K	Q	L	V	Q	A	F	L	S	C	L	L	T	T	A	F	I	Y	L	W	T	R	L	L	−66.216	Binder	Y	Y	Transmembrane	None	N/A
					*h*	*h*	*h*	*h*	*h*	*h*	*h*	*h*	*h*	*h*	*h*	*h*	*h*	*h*	*h*	*h*	*h*	*h*	*h*	*h*	*h*	*h*	*h*	*h*	*h*	*h*	*h*								
Other Bcl-2 proteins	Q9BXK5	Bcl-Rambo (Human)	E	G	K	S	I	L	L	F	G	G	A	A	A	V	A	I	L	A	V	A	I	G	V	A	L	A	L	R	K	K	−51.061	Binder	Y	Y	Transmembrane	None	N/A
											*h*	*h*	*h*	*h*	*h*	*h*	*h*	*h*	*h*	*h*	*h*	*h*	*h*	*h*	*h*	*h*	*h*	*h*	*h*	*h*									
Non-Bcl-2 proteins	P21397	MAOA (Human)	V	S	G	L	L	K	I	I	G	F	S	T	S	V	T	A	L	G	F	V	L	Y	K	Y	K	L	L	P	R	S	−46.097	Binder	Y	N	Transmembrane	None	N/A
							*h*	*h*	*h*	*h*	*h*	*h*		*h*	*h*	*h*	*h*	*h*	*h*	*h*	*h*	*h*	*h*	*h*	*h*	*h*	*h*												
	P27338	MAOB (Human)	G	L	L	R	L	I	G	L	T	T	I	F	S	A	T	A	L	G	F	L	A	H	K	R	G	L	L	V	R	V	−38.783	Binder	Y	Y	Transmembrane	None	N/A
						*h*	*h*	*h*	*h*	*h*	*h*	*h*	*h*	*h*	*h*	*h*	*h*	*h*	*h*	*h*	*h*	*h*	*h*	*h*	*h*														
	P33379	ActA (Listeria M)	N	H	T	T	L	I	L	A	M	L	A	I	G	V	F	S	L	G	A	F	I	K	I	I	Q	L	R	K	N	N	−39.027	Binder	Y	N/A	Transmembrane	None	N/A
				*h*	*h*	*h*	*h*	*h*	*h*	*h*	*h*	*h*	*h*	*h*	*h*	*h*	*h*	*h*	*h*	*h*	*h*	*h*	*h*	*h*	*h*	*h*	*h*	*h*	*h*	*h*									
	P80967	TOM5 (Yeast)	T	E	K	T	L	K	Q	A	A	Y	V	A	A	F	L	W	V	S	P	M	I	W	H	L	V	K	K	Q	W	K	−55.709	Binder	Y	N/A	Transmembrane	Fully resistant to sodium carbonate extraction and trypsin digestion	9217162
					*h*	*h*	*h*	*h*	*h*	*h*	*h*	*h*	*h*	*h*	*h*	*h*	*h*	*h*	*h*	*h*	*h*	*h*	*h*	*h*	*h*	*h*	*h*	*h*	*h*	*h*	*h*								
	P00167	Cb5 (Human)	S	S	S	S	W	W	T	N	W	V	I	P	A	I	S	A	V	A	V	A	L	M	Y	R	L	Y	M	A	E	D	−22.46	Sensor	Y	Y	Transmembrane	Detection of ER luminal side of Cb5 CTS by immunofluoresence	8947848
						*h*	*h*	*h*	*h*	*h*	*h*	*h*	*h*	*h*	*h*	*h*	*h*	*h*	*h*	*h*	*h*	*h*	*h*	*h*	*h*	*h*	*h*	*h*	*h*										

Residues predicted to form α-helix are indicated with the small letter h in purple below the residue [137]. For each CTS, the predicted transmembrane residues by web-server transmembrane predictor [38] and predicted tail-anchored sequences by TAMP finder [39] are specified where applicable (Y = Yes, N = No). Finally, negatively charged membrane-binding prediction based on free energy calculation was done using PMIpred [37].

**Table 2.** Summary of CTS-mediated interactions among Bcl-2 family members.

	Interactions	Method of detection	Functional consequence	PMID	References #
1	Homotypic Bax CTS-Bax CTS	1. ToxR-reconstitution in *E. coli* inner membrane to turn on RFP. 2. Split Venus-BiFC assay in HCT116 cells	Forming Bax dimer and expanding Bax oligomers-lining pores on MOM.	28028215	[18]
		Disulfide cross-linking of single-Cysteine Recombinant Bax by Copper Phenathroline (CuPhe)		27381287 26702098	[40] [41]
2	Homotypic Bak CTS-Bak CTS	Disulfide cross-linking of single-Cysteine Recombinant Bak induced by CuPhe	May involve in expanding Bak oligomeric complex after Bak dimerization step in Bak activation.	25744027	[42]
3	Bax CTS-Bcl-XL CTS	1. Competition dominant-negative ToxR assay in *E. coli* inner membrane to turn off RFP expression. 2. Competition split Venus BiFC assay to decrease Venus reconstitution	Inhibits Bax activation	28028215	[18]
		Single Cysteine cross-linking between recombinant Bax and Bcl-XL with the bifunctional BMH cross-linker		24616095	[43]
4	Bax CTS-Bcl-2 CTS	1. Competition dominant-negative ToxR assay in *E. coli* inner membrane to turn off RFP expression. 2. Competition split Venus BiFC assay to decrease Venus reconstitution	Inhibits Bax activation	28028215	[18]
5	Bak CTS-VDAC2	Blue Native-PAGE gel with radiolabelled Bak or Bak chimera which has Fis1 TMD incubated with isolated mitochondria from WT versus VDAC2^−/−^ MEF cells.	Inhibits Bak (auto)activation	20851889	[44]
6	Bok CTS-Mcl-1 CTS	Split Venus BiFC reconstitution	Inhibits and regulates ER-localized Bok activation from the mitochondrial outer membrane	33093207	[17]
7	Bim CTS-Bcl-XL/Bcl-2	1/ FLIM-FRET in BMK DKO cells expressing mCerulean3-fused Bcl-XL/Bcl-2 and Venus-fused Bim or BimΔCTS 2/Recombinant Bim for cell-free FRET assay for binding to Bcl-XL and iodine -NBD quenching assay to examine topography of Bim CTS binding site.	Double bolt-locking Bim to Bcl-XL and Bcl-2 causing resistance to displacement by BH3 mimetics	30860026	[14]
8	Puma CTS-Bcl-XL/Bcl-2	1/ FLIM-FRET in BMK DKO cells expressing mCerulean3-fused Bcl-XL/Bcl-2 and Venus-fused Puma or PumaΔCTS 2/Recombinant Puma for cell-free FRET assay for binding to Bcl-XL	Double bolt-locking Puma to Bcl-XL and Bcl-2 causing resistance to displacement by BH3 mimetics	37078707	[16]
9	Bim CTS-Bax	Recombinant protein for cell-free FRET assay with Bax	Bim CTS targets a secondary binding site on Bax in addition to Bim BH3 to efficiently activate Bax	31976859	[15]
10	Bok CTS-Bcl-2 CTS	Bimolecular split luciferase assay (Nanobit)	Inhibits Bok activation	N/A	[45]

## Canonical role of Bcl-2 CTSs in localizing proteins to subcellular membranes

The CTS enables Bcl-2 proteins to bind to specific subcellular membranes as CTSs often contain localization sequences for MOM, Golgi, and ER, as reviewed previously [13]. Moreover, CTSs of all multi-BH motif Bcl-2 proteins except for Bcl-2 A1 are predicted to have transmembrane domains enabling them to become fully inserted in the lipid bilayer (Table 1). On the other hand, BH3-only proteins apart from Bik [11] and Hrk [46] do not possess canonical transmembrane domains. Nevertheless, fusion proteins in which the CTS is attached to a normally cytoplasmic fluorescent protein such as GFP confirmed that the CTS sequences on most Bcl-2 family proteins are sufficient for both correct localization and membrane binding [35,47]. In addition to binding to membranes, localization is crucial for the apoptotic regulatory function of Bcl-2 proteins as different subcellular localizations can determine the response to various cell death stimuli [48–50]. For instance, the CTS of the anti-apoptotic protein Bcl-2 localizes the protein to both the mitochondria and ER membrane [50,51]. By replacing the CTS of Bcl-2 with that of cytochrome B5 (Cb5) or Actin assembly-inducing protein (ActA) from *Listeria* bacteria, Bcl-2 was specifically localized to the ER or mitochondria, respectively. ER-localized Bcl-2 protected cells from apoptosis induced by Myc overexpression [52], Staurosporine, BrefeldinA/Cycloheximide, Tunicamycin, and Thapsigargin [53], as well as by overexpression of Bax [54], but it did not protect cells from apoptosis induced by etoposide [49,52]. In contrast, mitochondria-localized Bcl-2 protected cells from apoptosis induced by all the stressors [49,55]. Another example of the importance of the CTS is an acquired mutation (P168A) in Bax that confers resistance of acute myeloid leukemia (AML) cells to the FDA approved Bcl-2 inhibitor, Venetoclax [56]. By competing for the canonical BH3-binding groove, BH3-mimetics such as Venetoclax and WEHI-539 either directly or indirectly release bound activated Bax from Bcl-2 or Bcl-XL, respectively [26] to activate apoptosis. Bax, unlike Bcl-2, is cytosolic in healthy cells [57,58]. Once activated, Bax binds to MOM [59,59] or other subcellular organelles such as ER [58] or the Golgi [61]. The acquired P168A mutation upstream of the Bax CTS impairs Bax binding to MOM [56,62]. However, other publications indicated that P168A Bax can still permeabilize liposomes and isolated mitochondria [63] or can be recruited to MOM in the presence of activated functional WT Bax after an apoptotic stimulus despite being unable to target the MOM when expressed alone [64]. These studies together paint a complex picture where the CTS plays a role in the proper localization of Bcl-2 family proteins thereby affecting the functions in apoptosis.

A main challenge in studying CTSs of Bcl-2 proteins is to differentiate their function in subcellular localization from their other functions in mediating protein-protein interactions. One common approach in studying the functions of CTSs is to make C-terminal deletion mutants at different truncating points [14–16]. Complete truncation of the CTSs typically causes Bcl-2 proteins to lose their normal subcellular localization and become cytoplasmic [14,16]. Therefore, if CTS-truncated Bcl-2 proteins do not interact with their binding partners, it would be unclear whether this is a result of losing CTS-mediated protein-protein interactions or losing the localization of proteins to subcellular membranes. Because of such complications, an alternative approach is required to study the effect of the CTSs on protein-protein interactions among Bcl-2 family proteins.

As mentioned above for Bcl-2, one alternative approach was replacing the CTS of Bcl-2 proteins with that of canonical tail-anchored proteins residing at the MOM (e.g. ActA or Monoamine oxidase A) or ER membranes (e.g. Cb5). This approach preserves the chimera Bcl-2 proteins binding to specific subcellular membranes in cells enabling experiments to examine the function of the membrane-bound Bcl-2 proteins in the absence of their corresponding CTS. While much useful data have been acquired with these mutants, the possibility exists of interactions between the swapped-in canonical tail-anchor sequence with themselves or with Bcl-2 family members further complicating how the results are interpreted. A second alternative approach is to replace the CTS with a C-terminal poly-Histidine sequence in the CTS-truncated recombinant Bcl-2 proteins that are then assayed using an *in vitro* system with MOM-mimicking liposomes [65]. Here, Nickel-chelating (NTA) lipids are incorporated into the lipid bilayer to recruit His-tagged Bcl-2 proteins to the liposome without using the endogenous CTS. This approach enabled studying Bak and Bok activation [66] as full-length Bak and Bok are difficult to purify as recombinant proteins due to their hydrophobicity. Nevertheless, this approach can induce auto-activation of Bak at concentrations higher than 100 nM [66]. Finally, making systematic mutations in the CTS might identify a mutation that impairs CTS-mediated protein-protein interactions but does not or only minimally affect binding to membranes [15,16]. To test this, fluorescence microscopy can be used to evaluate the co-localization between exogenously expressed fluorescence protein-tagged mutants with an altered or wild-type CTS and fluorescent dyes staining mitochondria (MitoTracker or TMRE) or ER membrane (BODIPY-Thapsigargin).

While mutating the CTSs as described above is useful to disrupt their function, these approaches do not directly show binding of the CTSs of Bcl-2 proteins to their binding partners. To show direct binding with CTSs, fusion proteins can be expressed in cells where the CTS and its potential binding partner are joined to proteins that function as sensors for protein-protein interactions. An example of this is the split Venus biomolecular fluorescence complementation (BiFC) system where each fragment of Venus is fused N-terminally to the CTSs of interest. Interaction of CTSs brings together the two non-fluorescent fragments to reconstitute a fluorescent Venus protein [18]. However, this technique has the risk of false positives as the two non-fluorescent fragments, due to their affinity for one another, can spontaneously associate or might not easily dissociate pushing the equilibrium towards the bound state. An alternative approach, used to detect interactions between the CTS of Bok and of Bcl-2, employs a split luciferase system named Nanobit in which the subunits called the large BiT (LgBiT) and the small BiT (SmBiT) were optimized to have low affinity for one another and are therefore better suited for studying protein complexes in membranes or with high dissociation rates [67,68]. Other techniques that have been used to directly demonstrate protein-protein interactions between CTSs include the ToxRed assay [18] and chemical cross-linking with recombinant Bcl-2 proteins that have a single Cysteine or Lysine in or near the CTS [14,15]. All these techniques require careful evaluation to ensure that the chimeric or modified Bcl-2 CTSs still localize to their respective subcellular membranes and that collisions are not confused with actual binding interactions.

### The CTSs of Bax, Bak, and Bok modulate pore-forming activity

Although binding to membranes by the three executioner proteins typically requires the hydrophobic CTS, the role(s) of the CTS differs between Bax, Bak, and Bok in the intricate, multi-step activation and oligomerization process. Activation of executioner proteins involves conformational changes in the monomeric inactive protein that result in partial unfolding and exposure of typically hidden hydrophobic surfaces [69–71]. These exposed hydrophobic regions, including BH3-binding sites and sometimes the CTSs, mediate interactions with other executioner proteins, anti-apoptotic proteins, and the lipid membranes of mitochondria or ER. Integration of the activated executioner proteins into the lipid bilayer occurs concurrent with or precedes oligomerization [69,72,73]. Thus, we will explore how CTSs of executioner proteins are involved in both the localization of these proteins at subcellular membranes and their oligomerization.

Bax, distinct from Bak and Bok, predominantly resides in the cytoplasm of healthy cells but undergoes continuous cycling between cytosol and the MOM [57,74–77]. This cycling, facilitated by the flexibility of the Bax CTS, is a direct result of an equilibrium between binding and displacement of the Bax CTS into and out of the canonical BH3-binding groove [78]. In the inactive cytoplasmic state, the equilibrium favors the Bax CTS occupying the canonical BH3-binding pocket of Bax, yet a subset of cytosolic Bax can bind peripherally to the MOM without activation [75,79]. Upon activation, Bax localization shifts towards the MOM, accompanied by the release of the Bax CTS from the canonical BH3-binding groove [78,80]. Consistent with the importance of Bax CTS insertion into membranes, and the function of Bax as an anti-oncogene, cancer-associated mutations of the Bax CTS (A183T or A183P, or truncating mutations at S184 and W188) [81] or phosphorylation of a key residue within the Bax CTS (S184) [82] diminished Bax binding to the MOM and the subsequent conformational changes required for Bax oligomerization. Furthermore, as discussed above, the acquired mutations P168A in Bax confers resistance in AML to the Bcl-2 inhibitor Venetoclax [56] by preventing Bax binding to membranes. As a result, activated P168A mutant Bax in AML cells displaced from Bcl-2 by Venetoclax cannot bind to and perforate the MOM. The resistance mechanism of the P168A mutation was hypothesized to be caused by a structural deformation that retains the mutated CTS in the canonical BH3-binding groove of Bax [62].

To induce the displacement of the Bax CTS, BH3 motifs of direct activator BH3-only proteins directly bind to the canonical BH3-binding pocket [71] as shown by X-ray crystallography or to another BH3-binding site on Bax known as the trigger site [83]. There is NMR data for Bax and a stapled Bim BH3 peptide [78] as well as cross-linking studies with full-length tBid and Bax [84] that support a mechanism involving the binding of the BH3 motifs of these proteins to the trigger site located across helix 1 and 6 near the N-terminus of Bax, essentially on the opposite side to the canonical BH3-binding groove. Such binding has been proposed to produce an allosteric change in the core of Bax, resulting in the mobilization of the Bax CTS and Bax BH3, and the subsequent exposure of the canonical BH3-binding groove of one Bax molecule to the BH3 motif of another Bax or of a direct-activator protein. While compelling, this model for Bax activation remains contentious because binding of the Bax CTS to the Bax hydrophobic groove is dynamic thus, it is not clear that binding to the trigger site is always required to activate Bax. Binding of a BH3 motif to the canonical BH3-binding groove on Bax induces further conformational changes, specifically unlatching of an amino terminal region that promotes dimerization and subsequent homo-oligomerization of Bax [71,78]. Bax conformation changes, dimerization and oligomerization apparently take place on the membrane, immediately after insertion of the CTS into the lipid bilayer [69,73,85]. However, there is also evidence that a fraction of monomeric Bax forms dimers prior to insertion into the MOM, with oligomerization of the preformed dimers taking place after insertion into the membrane [86–88]. In either case, by making the BH3-binding groove less accessible to BH3 motifs, binding of Bax CTS to the canonical BH3-binding groove may serve as an inhibitory mechanism preventing spontaneous activation. As a result, an activator BH3-only protein must have sufficient affinity to displace the Bax CTS in order to bind the canonical BH3-binding groove [89–91]. Supporting this hypothesis, fluorescence polarization assays with FITC-labelled stapled Bid BH3 peptides and recombinant Bax demonstrated a 3-fold increase in binding affinity of the Bid BH3 peptide to CTS-truncated Bax compared with full-length Bax in solution [89]. This model of Bax inhibition is further substantiated by the loss of pore-forming activity of Bax in the presence of the Bim BH3 when the Bax CTS is locked in the canonical BH3-binding pocket by a disulfide bond tether (A112C-V177C) [78]. Overcoming this inhibitory mechanism may require binding to an allosteric site on Bax, as proposed by the ‘trigger site’ model, either prior to or simultaneously with binding of the BH3 motif of a direct activator to the canonical BH3-binding groove of Bax. Binding to both the trigger site and the canonical BH3-binding groove of Bax might be possible for the BH3-only protein Bim that binds to and activates Bax using both the Bim BH3 and the Bim CTS [15]. As discussed previously, release of the Bax CTS from the canonical BH3-binding groove also results in Bax binding to the MOM whereupon, Bax monomers organize into dimers that then oligomerize in the membrane and permeabilize the lipid bilayer [78,79]. It is worth noting that Bax activation can also be induced by small molecules [92], heat [93], and certain lipids [94]. In these cases, the role of the trigger site in Bax activation is not yet studied.

**Figure 1.**
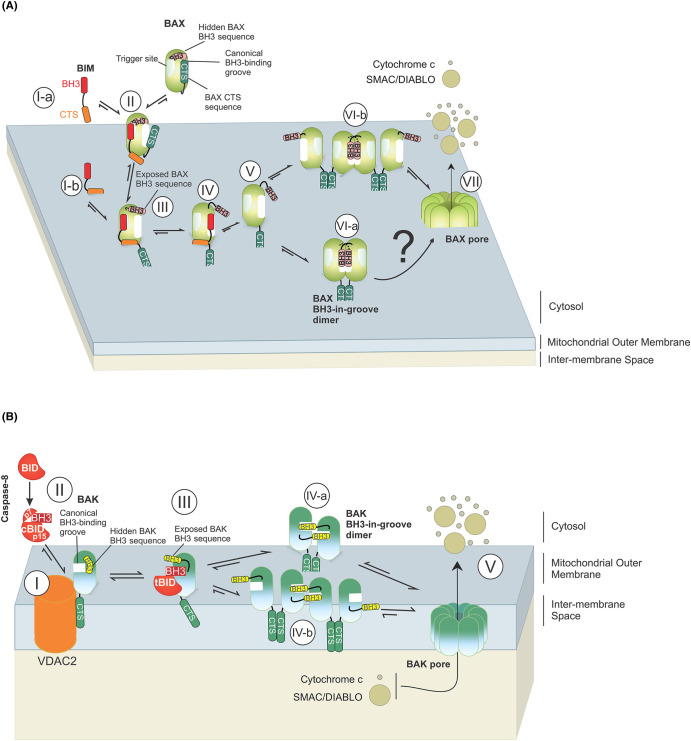
The CTSs of Bax and Bak regulate their pore-forming functions. (**A**) The CTS and BH3 of Bim are required for Bim to bind Bax in the cytosol [15] (l-a) and on the MOM (l-b). It is unknown where the Bim CTS binds on Bax. Binding of Bim BH3 to the trigger site of Bax induces conformational changes that displace the CTS of Bax from the canonical BH3-binding groove enabling Bax to bind to the MOM (II) and expose the BH3 of Bax [78] (III). As a result, the canonical BH3-binding groove of Bax is accessible for binding a Bim BH3 sequence. This leads to further conformational changes in Bax (core-latch domain separation [71]) (V) enabling Bax to dimerize with the BH3 of one Bax inserted into the canonical BH3-binding groove of another Bax (BH3-in-groove model [96]) (VI). The Bax CTSs can interact with each other to form an additional dimerization interface either in the Bax BH3- in-groove dimer itself [40] (Vl-a) or between monomers from two different BH3-in-groove Bax dimers [41] (Vl-b) to form higher-order Bax oligomers. Finally, Bax oligomers form large ring-like pores on the MOM releasing pro-apoptotic factors such as cytochrome c and SMAC/Diablo from the intermembrane space into the cytosol [21,97] (VII). It remains unclear that binding to the trigger site is always required and in which circumstances BH3 sequences may compete directly with CTS binding to activate Bax. (**B**) The Bak CTS is required for Bak to interact with Voltage-dependent Anion Channel 2 (VDAC2) which keeps Bak in its inactive state [44] (I). Caspase 8 cleaves Bid to generate (cleaved) cBid in which the two fragments (p7 and p15) are held together by hydrophobic interactions [98,99] (II). The truncated C-terminal p15 fragment of cBid (tBid) inserts into the MOM where it binds to Bak displacing it from VDAC2 thereby activating Bak [100] (III). Activated Bak forms dimers via both the BH3 and CTS sequences where BH3 of one Bak is inserted into the canonical BH3-binding groove of another Bak [101] and the Bak CTSs dimerize [42] (IV). Similar to Bax, it is possible that Bak CTSs dimerize Bak from different Bak dimers (IV-b) as well as Bak from within the same BH3-in-groove dimer (IV-a). Interactions between Bak CTSs of different Bak dimers (IV-b) would enable formation of high-order oligomers that permeabilize the MOM [102] (V).

On the MOM, binding of the BH3 motif of an activated Bax into the BH3-binding groove of the second protomer is a well-established mechanism of Bax dimerization [41,95,96]. In a lipid environment, the Bax CTS has also been shown to participate in the dimerization interface between two Bax monomers [18,40] (Figure 1A). Moreover, in the absence of membranes, dimerization of CTS-truncated Bax is greatly reduced [36]. Nevertheless, in cells, upon activation by tBid other domains in Bax can oligomerize CTS-deleted Bax [103]. The formation of Bax dimers via the CTS may establish a separate dimerization interface, facilitating the formation of higher-order Bax oligomers in the MOM which can aid in the expansion of Bax pores [41] (Figure 1A). This possibility is supported by observations that the CTS of Bax, when expressed independently, can undergo homo-oligomerization/dimerization on the MOM, as demonstrated in live cells through BiFC assays [18]. Whereas, in control experiments, the mitochondrial membrane binding tail anchor of TOM20 did not dimerize or oligomerize [18]. The soluble Bax monomer has been reported to also form unproductive auto-inhibited dimers when concentrated to above 5 mg/ml [104]. In this inactivated dimeric form, the Bax CTS of the first protomer engages with the trigger site of the second protomer thereby blocking the trigger site of the second protomer as well as hindering the mobilization of Bax-CTS of the first protomer.

In contrast with Bax that, at steady state in most cells, is primarily cytoplasmic and Bok that is predominantly localized at the ER [105], Bak is located almost exclusively at the MOM, even in its inactive form [106]. This is attributed to the Bak CTS (helix α9) having a higher hydrophobic residue content compared with the Bax CTS [70] causing Bak to be constitutively embedded in the lipid bilayer of the MOM [34,42,106]. Deletion of the CTS abolishes Bak-mediated cell death in response to UV exposure or etoposide treatment in mouse embryonic fibroblast (MEF) cells [107]. However, previous studies using recombinant soluble CTS-truncated murine Bak (residue 16–184) fused with a C-terminal hexa-histidine tag (BakΔC-His) that can bind to large unilamellar vesicles (LUVs) containing Ni^2+^-NTA lipids showed that the Bak CTS is not required for Bak to permeabilize liposomes as long as the BakΔC can bind to the membrane [108]. Importantly, the BakΔC-His used in this study is auto-active at nanomolar concentrations such that it can efficiently permeabilize the LUVs with Ni^2+^-NTA in the absence of cleaved Bid. However, the BakΔC-His used in the study was missing the first 16 amino acids at the N-terminus, known as the N-segment, which may serve a role in keeping Bak non-activated by preventing the formation of a Bak dimer-interface [109]. Therefore, auto-activation of BakΔC-His might not resemble how wild-type Bak becomes activated. Another study showed that CTS-truncated Bak can form pores on Giant Unilamellar Vesicles in the presence of cBid but requires a high concentration of Bak (300 nM) and a longer incubation time than that for Bax [110]. Overall, these studies imply that the primary role of the Bak CTS is for localizing Bak to the MOM where Bak can readily become activated. Nevertheless, the Bak CTS was shown to interact with other Bak CTSs once Bak becomes multimerized. Using digitonin-permeabilized cells exogenously expressing Bak mutants containing cysteine residues in the Bak CTS, the addition of tBid resulted in cross-linking of the Bak CTS by disulfide bonds formed between the introduced cysteine residues (Figure 1B) [42]. In addition, upon introducing other cysteine residues in the Bak BH3 and the canonical BH3-binding groove in addition to the cysteine in the Bak CTS for disulfide cross-linking, higher molecular-weight bands corresponding to high-order oligomers of Bak were observed. These results indicate a potential involvement of the CTS in the formation of Bak oligomers.

However, the cross-linking data could be explained as resulting from collisions of Bak CTS in the lipid environment upon oligomerization of Bak rather than the result of a functional interaction that is required for Bak oligomerization [42]. This study did examine the functional effect of deleting Bak CTS in etoposide-induced cell death by expressing a chimeric Bak mutant in which the Bak CTS was replaced with CTSs of proteins targeting to mitochondria such as Fis1 and MOA (Monoamine Oxidase A). Bak with Fis1 CTS but not MOA CTS can efficiently kill cells after etoposide treatment, causes cytochrome C release, and can become disulfide-cross-linked when tBid is added to mitochondria isolated from cells expressing this Bak mutant [42]. This shows that the CTS of a mitochondrial protein upon substituting in for Bak CTS can be sufficient not only for targeting Bak to the MOM but also for dimerizing and forming Bak pores upon Bak activation by tBid.

The CTS:CTS interface of wild type Bak that resulted upon tBid addition did not require binding of the Bid BH3 domain to the canonical BH3-binding groove of Bak [42]. Mutations in this groove or blocking the groove with an anti-Bak BH3 antibody did not hinder the disulfide cross-linking of the Bak CTSs in the presence of tBid in the membrane fractions extracted from Bax^−/−^ Bak^−/−^ MEF cells expressing the single-cysteine Bak CTS mutants. This intriguing finding highlights a distinct mechanism for Bak activation and its interaction with tBid, independent of the canonical BH3 binding groove [42]. Altogether, these studies show that homo-dimerization of the Bak CTS may not be absolutely required for Bak oligomerization but could be a consequence of dimerization or oligomerization of Bak monomers following their activation. It remains to be tested if dimerization of the Bak CTS contributes to controlling the size of Bak pore, similar to the model proposed for the Bax CTS in expanding Bax pore.

Bok is the least studied executioner protein of the Bcl-2 family, and consistent with the indirect activation model for Bax/Bak, Bok has been shown to be constitutively active. Bok is proposed to be regulated both by binding anti-apoptotic proteins and by changes in stability as Bok has a short half-life and is rapidly degraded in cells [111]. Therefore, our understanding of the Bok CTS is limited. We do know that the Bok CTS localizes the protein predominantly to the ER, Golgi, and associated membranes [104]. Unlike Bax and Bak, recombinant Bok lacking its CTS (ΔCTS) is auto-active and at concentrations above 10 nM permeabilized MOM-like LUVs in the absence of an activator BH3-only protein [23]. However, the presence of cBid has been reported to enhance the LUV permeabilizing activity of BokΔCTS [112]. Targeting recombinant BokΔCTS to LUV containing Ni^2+^ modified lipids using a C-terminal His-tag fused to BokΔCTS, also results in permeabilization of LUVs [111]. In the same experiment, full-length Bax and truncated BakΔCTS with a C-terminal His-tag, bound to LUV with Ni^2+^-NTA modified lipids, but remained inactive until the addition of cBid. These results support the hypothesis that, unlike Bax and Bak, Bok can auto-activate in the absence of BH3-only direct activator proteins. Intriguingly, at 100 nM recombinant BokΔCTS alone is not sufficient to permeabilize mitochondria isolated from Bax/Bak double knockout (DKO) MEF cells [112] but can permeabilize mitochondria isolated from DKO HCT116 (Human Colorectal Carcinoma) cells in a dose-dependent manner from 200 nM to 5 µM [23]. In live cells, transiently expressed GFP-BokΔCTS fusion protein has a slight reduction in cell-killing activity in DKO HCT116 compared with GFP-full-length Bok, as measured by Annexin V positivity [23,34]. Although the involvement of the Bok CTS in cell death is still unclear, these studies suggest that the Bok CTS is not required for its pore forming function but rather for localization of Bok to its target intracellular membrane.

### The CTSs of Bax, Bak, and Bok interact with anti-apoptotic proteins

Activated Bax and Bak are at the MOM where anti-apoptotic proteins heterodimerize with monomeric Bax or Bak thereby inhibiting them [43,113]. This binding competes directly with the homotypic interactions of Bax/Bak and prevents homodimer/oligomer formation in the MOM, thereby inhibiting cell death initiation [5]. One well-studied binding interface between Bax/Bak and anti-apoptotic proteins is the binding of the Bax or Bak BH3 motif to the canonical BH3-binding groove of Bcl-XL, Bcl-2 or Mcl-1 that is composed of the BH1, BH2, and BH3 motifs [43,114–116]. The BH3 motifs in helix α2 of Bax and Bak are amphipathic and are typically concealed in the hydrophobic core of inactive Bax/Bak [90]. Therefore, as described in the previous section, these executioner proteins must undergo an activation-induced conformational change that exposes the BH3 motif to bind anti-apoptotic proteins [26,43,114,117,118].

Another binding interface on Bax for binding to anti-apoptotic proteins is the trigger site. This site can be cross-linked to the BH4 motif (α1) of full-length Bcl-XL [43] or Bcl-2ΔCTS [114] suggesting a possible mechanism of negative regulation. Bak, on the other hand, is not known to possess a trigger site and activation of Bak is reported to be either constitutive or to be due to binding of the BH3 motif of BH3-only direct activator proteins to the canonical BH3-binding groove [70]. Adding more complexity to this model, accessibility of the canonical BH3-binding groove in Bak and thereby Bak activation are further regulated by (1) a structural constraint that involves the N-terminal BH4 sequence (α4) interacting with the hydrophobic core (α2-α5) of Bak [119] or (2) interaction of Bak with Voltage-dependent anion channel 2 (VDAC2) on the MOM [44,100,120,121]. VDAC2 also plays a role in the recruitment of Bak to mitochondria [120]. It is unclear how direct activator BH3-only proteins or other mechanisms overcome these regulatory binding interactions to activate Bak.

**Figure 2.**
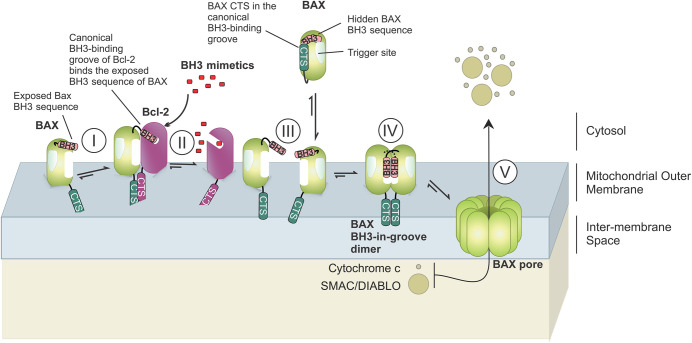
The CTS of Bax binds to the CTS of Bcl-2. The Bax CTS interacts with the CTS of Bcl-2. Upon activation, Bax binds to the MOM but becomes inhibited by binding to the anti-apoptotic protein Bcl-2, and by analogy Bcl-XL (not shown here), such that the exposed Bax BH3 becomes sequestered in the canonical BH3-binding groove of Bcl-2 [114] (I). The CTSs of Bax and of Bcl-2 bind and form an additional interface in this heterodimer [18]. Upon treatment with BH3-mimetics like ABT-263, targeting the BH3-binding groove of Bcl-XL/Bcl-2, activated Bax might be released (II) from the anti-apoptotic proteins to bind to another MOM- bound Bax (III), thereby forming Bax dimers (IV) and oligomers which permeabilize the MOM (V). It is currently unclear whether BH3-mimetics directly displace activated Bax from Bcl-2 or prevent the formation of new Bax-Bcl-2 heterodimeric complexes. For simplicity, only one pathway of Bax dimerization and oligomerization is shown. Both potential pathways for Bax oligomerization are shown in Figure 1.

In addition to binding mediated by BH3 motif and the trigger site, the CTSs of Bax and of Bcl-XL are in proximity with one another and thus can be cross-linked in MOM-like liposomes using single cysteine mutants and the cysteine-reactive BMH cross-linker [43]. Further supporting this hypothesis, the Bax CTS interacted with the CTS of both Bcl-XL and Bcl-2 in *Escherichia coli* using the ToxRed system and in a competition BiFC assay in HCT116 cell lines [18] (Figure 2). Moreover, in an *in situ* proximity biotinylation assay in HCT116 cells, BirA fused to the CTS of Bax (BirA-Bax-CTS) biotinylated endogenous Bcl-XL, whereas the mitochondria-localized negative control, TOM20, was not labeled [18]. Unfortunately, this study did not determine if the CTSs of Bax or of Bcl-XL/Bcl-2 are necessary for anti-apoptotic proteins to inhibit Bax activation directly. Such studies are technically demanding due to the induction of apoptosis by Bax. To address the issue they demonstrated that exogenous expression of just the CTS of either Bcl-2 or Bcl-XL reduced the activation of caspase 3 and 7 that resulted from exogenous expression of the Bax CTS in HCT116 cells [18]. How well the CTSs in isolation recapitulate interactions of the full-length proteins is unclear. Thus, it remains possible that once partially embedded into the MOM [41], Bax CTSs collide with, rather than bind to, Bcl-XL CTSs as a result of them being brought close together by the hetero-dimerization of the cytoplasmic parts of Bax and Bcl-XL (i.e. effect rather than cause). Additional support for direct binding of CTSs includes the recent demonstration that the Bok CTS interacts with the CTS of Bcl-2 in Hek293 cells using a bimolecular split luciferase assay [45]. Furthermore, Bcl-2-dependent inhibition of exogenous Bok induced apoptosis in DU145 cells was dependent on an interaction with the Bok CTS. Accordingly, apoptosis induced by exogenous expression of a Bok chimeric mutant in which the Bok CTS was replaced with the Bax CTS was not significantly inhibited by Bcl-2. In contrast with Bax and Bok, there have been no studies examining the interaction(s) between the Bak CTS and Bcl-2 family anti-apoptotic proteins. However, the CTS of Bak was shown to be necessary for interaction with VDAC2 and for maintaining Bak in an inactive high molecular mass complex on the MOM [44] (Figure 1B). Collectively, these studies suggest several potential roles of interactions mediated by CTSs in restraining Bax/Bak activation.

Similar to the interactions with Bcl-XL and Bcl-2 described above, Mcl-1 was identified using a proximity labeling assay as interacting with Bok [122]. This interaction was shown to require the CTS of Bok by experiments demonstrating that Bok, but not BokΔCTS, co-precipitated with Mcl-1. Additionally, an interaction between the CTSs of Bok and Mcl-1 was demonstrated using a BiFC assay with the split Venus system [17]. The assay also revealed that Bok CTS/Mcl-1 CTS heterodimers are localized to the mitochondria. This contrasts with observations that Bok localizes to the ER, Golgi, and associated membranes [105], suggesting that Bok might relocate from the ER/Golgi to the mitochondria in the presence of Mcl-1 due to their interactions at the CTSs. Moreover, when the Bok CTS and Mcl-1 CTS were co-expressed in Hela cells, there was an increase in the number of ER-mitochondria contact sites, also known as mitochondria-associated ER membranes (MAMs). This finding supports the hypothesis that the Mcl-1 CTS somehow recruits the Bok CTS to the MOM. Furthermore, cancer-associated mutations in the CTS of Mcl-1, such as A339T and L384V, were found to enhance the interaction between the Bok CTS and Mcl-1 CTS [17]. These mutations also increased Mcl-1 mediated suppression of Bok-mediated cell death. Together these results suggest there is a novel apoptosis regulatory mechanism involving the CTSs of Mcl-1 and the pro-apoptotic protein Bok. However, more detailed studies using full-length proteins in live cells are required to confirm that binding is direct and increases the localization of Bok to the mitochondria.

### The CTS of BH3-only proteins Bim and Puma bind to anti-apoptotic Bcl-2 proteins

**Figure 3.**
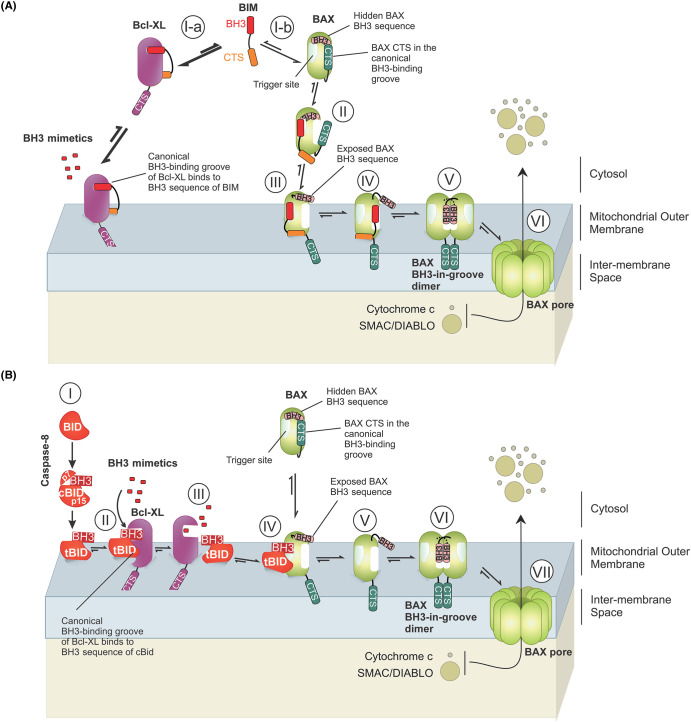
The CTS of Bim contributes to binding to anti-apoptotic proteins and Bax. (**A**) Bim BH3 and CTS sequences strongly bind to Bcl-XL in solution and at the MOM (thick arrows) ensuring that Bcl-XL outcompetes Bax for binding to Bim [14] (l-a). The Bim BH3 binds to the canonical BH3-binding groove of Bcl-XL [126], whereas the binding site for Bim CTS on Bcl-XL is unknown. The two binding sequences create a double-bolt lock that prevents BH3-mimetics from displacing Bim from the anti-apoptotic proteins. Bim that is not bound to Bcl-XL can bind to and activate Bax (l-b) as described in Figure 1. (**B**) In contrast to Bim, BH3-only protein Bid requires prior activation by caspase-8 mediated cleavage to form cBid (I). The p15 fragment of cBid (tBid) binds to Bcl-XL on the MOM via a BH3-dependent mechanism (II). Therefore, unlike Bim, tBid can be displaced by BH3-mimetics from the anti-apoptotic proteins [14] (III). The displaced tBid can then bind to Bax (or Bak) (IV) inducing conformational changes that expose Bax BH3 (V). This results in Bax dimerization (VI) and oligomerization (VII) on the MOM. For simplicity, only one Bax oligomerization pathway is shown.

BH3-only proteins localize to the MOM or ER by their hydrophobic CTSs [12,16,123]. While it is evident that the CTSs of BH3-only proteins enhance binding to anti-apoptotic proteins, the mechanism remains obscure. This is in part because, except for Bid [99], BH3-only proteins are intrinsically disordered. The unstructured BH3 sequences, as modeled using peptides, bind to anti-apoptotic proteins with affinities ranging from low nanomolar [124] to micromolar [125] depending on the length of the peptides [19]. These studies employed different binding assays and very few used full-length BH3-only proteins to measure binding affinities to anti-apoptotic proteins [19]. Using the hydrocarbon cross-linking (stapling) strategy to improve the stability and α-helicity of BH3 peptides, binding of Bid, Bim, and Bad BH3 stapled peptides to Bcl-XL had a dissociation constant (*K_d_*) ranging from 100–400 nM [89]. In contrast full-length Bim binds to Bcl-XL with an apparent *K_d_* of 3 nM in the presence of mouse liver mitochondria while truncating the Bim CTS results in a 10-fold decrease in the binding affinity [14]. Thus, while the direct binding of the BH3 motif is essential for BH3-only proteins to inhibit and be inhibited via mutual sequestration by multi-BH motif anti-apoptotic proteins, it is likely that additional interactions are required for BH3-only proteins and their binding partners to achieve biologically meaningful binding affinities. Recent findings suggest that the CTS of the BH3-only protein Bim functions as a secondary binding site for both Bcl-XL and Bcl-2 [14]. Bim binding to anti-apoptotic proteins at two sites creates a ‘double-bolt lock’ that prevents displacement of Bim from anti-apoptotic proteins by BH3-mimetic drugs (Figure 3A). Similarly, another BH3-only protein, Puma, can also ‘double-bolt lock’ to both Bcl-XL and Bcl-2 [16]. These ‘double-bolt locked’ protein complexes may have a sufficiently low off-rate that BH3-mimetics cannot compete to disrupt pre-existing BH3-only protein-anti-apoptotic heterodimers but may still prevent the formation of new protein complexes. Unlike these interactions with anti-apoptotic proteins the ‘double-bolt lock’ model is most likely not applicable for binding of Bim or Puma to Bax or Bak where the binding induces pronounced conformational changes in Bax/Bak that result Bax/Bak activation and oligomerization. The data supporting the double-bolt lock mechanism cannot be observed unless full length proteins are used. However, binding of the full-length Bcl-2 proteins to membranes is not required. Furthermore, the results were recapitulated in measurements in live cells using FLIM-FRET, thereby substantiating that for many Bcl-2 family proteins the interactions of full-length recombinant proteins are necessary for and accurately represent the interactions of membrane-inserted proteins in live cells.

Although these studies did not identify the binding sites of the CTSs of Bim or Puma on the anti-apoptotic proteins, they provided compelling evidence for the functional importance of the additional binding interactions. Using FRET-based protein-protein binding assays in cell-free systems with purified recombinant proteins direct binding was demonstrated. In addition, CTS dependent augmentation of binding was observed using FLIM-FRET in live mammalian cell systems for exogenously expressed proteins. These experiments showed that compared with full length proteins, truncated mutants of Bim and Puma lacking the CTS have lower affinity interactions with antiapoptotic proteins and were displaced by BH3-mimetics that inhibit Bcl-XL and Bcl-2. In contrast, binding of the sensitizer BH3-only protein, Bad and the active cleaved form of the activator BH3-only protein Bid (tBid) to Bcl-XL and Bcl-2 was sensitive to displacement by the BH3-mimetics ABT-263 [14,127] (Figure 3B). While Bad requires a CTS to target mitochondria [128], there is no evidence the Bad CTS binds to anti-apoptotic proteins. On the other hand, Bid does not have a well-characterized membrane-binding CTS [129,130] but does have a C-terminal region that contributes to localizing Bid to the MOM [123]. After cleavage of Bid by caspase 8, the cleaved Bid (p7 and p15 fragments) bind to membranes whereupon p7 separates from p15 (tBid). The latter binds to membranes via the stretch of amino acids from 103 to 162 of Bid, consisting of helices H4, H5 and H6 and undergoes further conformational changes on the MOM to bind Bcl-XL [131] or to activate Bax [99] or Bak [132]. Therefore, it is likely that tBid binding to anti-apoptotic proteins is mediated solely by the BH3 motif, explaining why it is displaced by BH3-mimetics. Noxa, another BH3-only protein that binds to the anti-apoptotic proteins Mcl-1 and A1 [133,134], has a non-canonical tail-anchor CTS that targets Noxa to the MOM [123]. Intriguingly, the Noxa CTS was reported to have a degron sequence that directs not only Noxa but also its protein-binding partner Mcl-1 to a ubiquitin-independent degradation pathway, thereby regulating the stability of the heterodimer [135]. Altogether, these studies may have significant implications for the use of BH3-mimetics in cancer treatment, as these drugs may not kill cancer cells in which Bim or Puma are held in check by sequestration with anti-apoptotic proteins.

One important, yet unclear, mechanistic detail regarding the function of the CTS in BH3-only proteins is whether high affinity binding to anti-apoptotic proteins is separate from membrane binding. Site-directed mutagenesis scanning of the Bim CTS revealed that an L185E mutation resulted in reduced localization of Bim to the mitochondria and displacement of Bim from Bcl-XL by the BH3-mimetic ABT-263 [14]. Similarly, in Puma CTS binding to Bcl-XL, Leucine 174 and Proline 180 contribute to resistance to displacement by BH3-mimetics and are necessary for Puma localization to the ER membrane [16]. These examples suggest that the membrane binding function of the CTS of Bim and Puma may be intertwined with their binding to multi-BH motif proteins.

The importance of membrane localization in conferring resistance of Bim and Puma to displacement from Bcl-XL by BH3-mimetics was first demonstrated by Bioluminescence Resonance Energy Transfer proximity measurements [26]. The truncation mutant, Bcl-XLΔCTS, which is primarily cytoplasmic, bound to Puma and Bim in live cells. However, these interactions were disrupted by low concentrations of the BH3-mimetic WEHI-539 (100 nM for Puma and 1 µM for Bim). The same concentrations of BH3-mimetic had no effect on predominantly mitochondria and/or ER-localized full-length Bcl-XL bound to Bim or Puma. Therefore, it was hypothesized that membrane binding serves as the primary driver of BH3-mimetic resistance in cancer cells. However, studies with purified full-length Bcl-XL together with full-length Bim and Puma, demonstrated that in the absence of a membrane, Bim and Puma binding to Bcl-XL was largely resistant to displacement by the BH3-mimetic ABT-263 [14,16] (Figure 3A). Therefore, while binding to membrane likely contributes to the apparent high affinity of the interactions of Bcl-XL with Bim and Puma, the CTSs of these BH3-only proteins are also important for resistance to displacement by BH3-mimetics. The site(s) of these interactions are yet to be determined. Thus, further studies are needed to examine the binding interface between the CTSs of BH3-only proteins and the corresponding binding sites on anti-apoptotic proteins.

### The CTS of the BH3-only protein Bim contributes to binding to and activation of Bax

The Bim CTS is necessary for both high affinity binding of Bim to the executioner protein Bax in the absence of a membrane (*K_d_*_ _= 60 nM for full-length Bim and >>1000 nM for BimΔCTS) and for the activation of Bax [15]. In control experiments binding of Bim to membranes by replacing the CTS with a heterologous CTS sequence derived from monoamine oxidase (MAO) that binds to mitochondrial membranes resulted in a chimera ‘BimΔCTS-MAO’ that was unable to activate Bax. Nevertheless, BimΔCTS-MAO retained high-affinity binding to Bax in the presence of liposomes or isolated mitochondria confirming the binding to membranes improved the apparent affinities. Mutagenesis experiments revealed that hydrophobic residues L129 and I132 within the Bim CTS were required for both activation of Bax and for binding to membranes [15]. Both Bim mutants L129E and I132E retained relatively high binding affinity for Bax in the absence of membranes (*K_d_* < 80 nM). Consistent with the importance of the CTS region, much higher concentrations of Bim BH3 peptides, whether stapled or un-stapled, are required to elicit Bax activation [89]. Taken together, these data suggest that the CTS of Bim is a multifunctional sequence that not only binds to membranes but contributes to double bolt-locking of Bcl-XL and efficient activation of Bax (Figure 3A). It will be important to determine whether the CTS of the BH3-only protein Puma is required not only for Puma to bind to intracellular membranes [16] but also for Bax binding and activation. If so, we speculate that Bim and Puma CTSs will bind to different sites on Bax and Bcl-XL, given the inherent differences in their sequences. Overall, the Bim CTS contains more hydrophobic residues than the Puma CTS and is predicted to have higher affinity for negatively charged membranes (Table 2). Proline, an alpha-helix breaker amino acid, also appears more frequently in the Puma CTS than in the Bim CTS thereby shortening the predicted alpha helix in the Puma CTS. Furthermore, the Puma CTS has more charged residues including negatively charged residues towards the C-terminal end of the protein. We hypothesize that these differences in the composition of amino acids would confer different protein binding properties to the Bim CTS and the Puma CTS.

**Figure 4.**
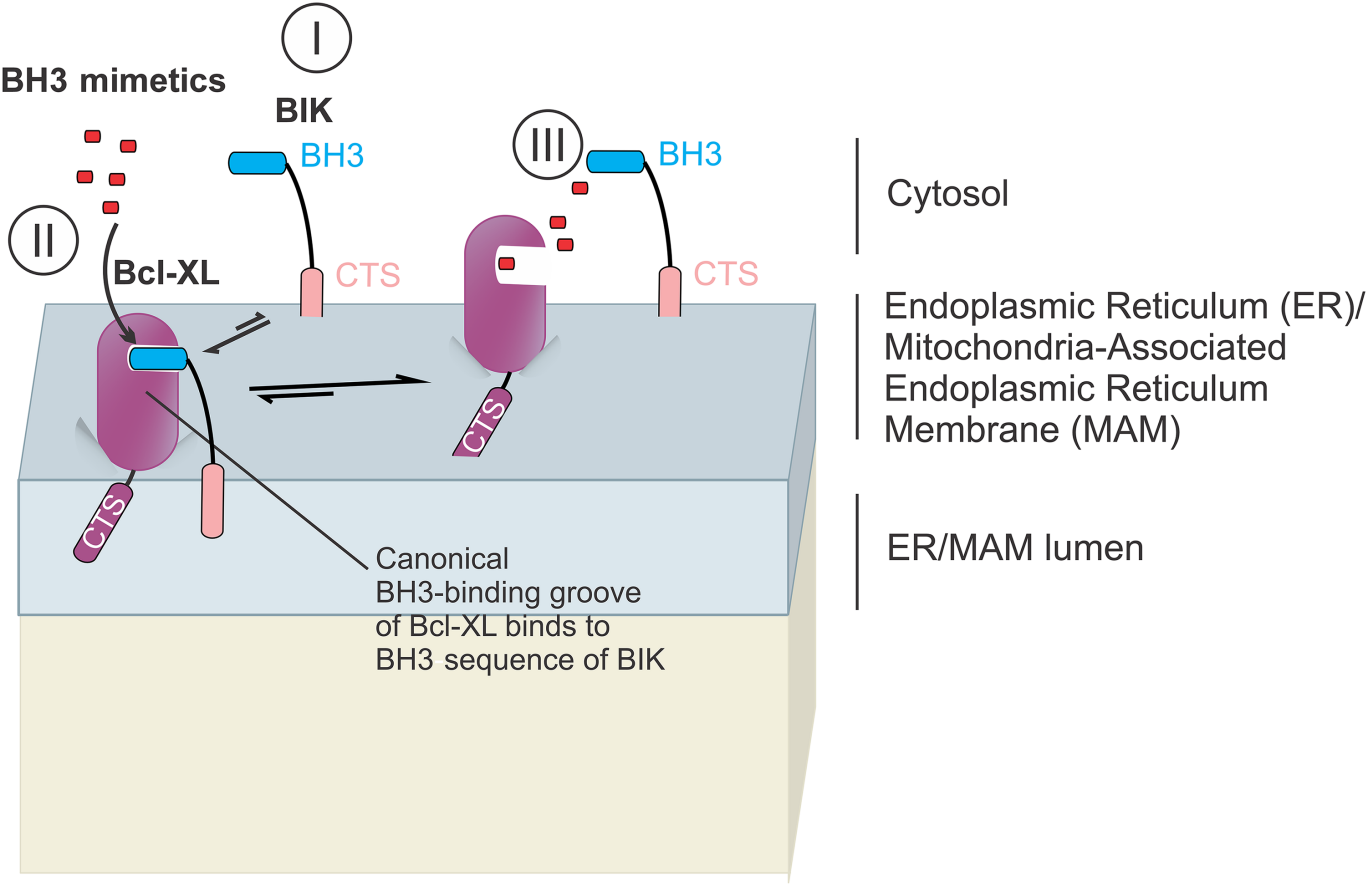
The CTS of BIK is a canonical tail anchor sequence that localizes BIK to the ER or the MAM but does not confer resistance of BIK to displacement by BH3-mimetics from Bcl-XL. BIK is inserted into the ER or MAM via the canonical tail anchor CTS [123,136] (I). BIK binds to Bcl-XL solely via the BIK Bh3 sequence binding to the canonical BH3-binding groove of Bcl-XL (II) and Bcl-2 (not shown) and does not bind to Bax or Bak. BH3-mimetics compete with the BIK BH3 sequence for the binding site on Bcl-XLthereby displacing BIK [127] (III).

In contrast with Bim and Puma, truncating the CTS of Bik, another BH3-only protein, abolished binding to the membranes in cells but did not affect its pro-apoptotic function in multiple cell types [136]. Therefore, it appears that the Bik CTS functions only as a canonical tail anchor required for the localization of Bik to the ER and the MAM [136] and does not confer resistance to BH3-mimetic displacement from Bcl-XL [127] (Figure 4). Thus, CTS-mediated membrane binding is not always sufficient to increase binding affinity nor is it required for pro-apoptotic function of all BH3-only proteins.

## Concluding remarks

Although many of the binding interactions between Bcl-2 family members that regulate apoptosis have been identified, recent data suggest that the molecular mechanisms involve more than the BH3 motifs. It is now clear that the membrane-binding CTSs of Bcl-2 family members contribute in several different ways to regulate protein-protein interactions between members of Bcl-2 family proteins. The simplest function of the CTS is binding to membranes without changing pro-apoptotic activity, as seen for BIK. The most complex scenario is exemplified by Bim and Puma where the CTS contributes to localization, membrane binding, inhibition of anti-apoptotic proteins and activation of pro-apoptotic proteins. We speculate the binding interface between CTSs of Bim and Puma and anti-apoptotic proteins could be potential drug targets as disrupting these binding sites would enable BH3-mimetics to efficiently displace Bim and Puma from the anti-apoptotic proteins and activate Bax or Bak to kill cancer cells.

The interactions of the CTS sequences of multi-domain pro-apoptotic proteins also have functional consequences that determine how cells respond to apoptotic stimuli, ultimately influencing cell fate. The CTSs of Bax, Bak, and Bok not only facilitate binding to the mitochondrial and ER membranes where they exert their pro-apoptotic functions but are also involved in the oligomerization of these pore-forming proteins necessary for permeabilizing the MOM. Additionally, the CTSs of Bax and Bok are targets for binding by anti-apoptotic proteins, such as Bcl-XL, Bcl-2, and Mcl-1, that inhibit oligomerization and promote cell survival. Therefore, understanding the molecular mechanism(s) of CTS binding interactions will provide new insights that may inform the development of future therapeutics.

## Abbreviations

AML: acute myeloid leukemia
BiFC: biomolecular fluorescence complementation
CTS: C-terminal sequence
DKO: double knockout
ER: endoplasmic reticulum
LUV: large unilamellar vesicle
MAM: mitochondria-associated ER membrane
MEF: mouse embryonic fibroblast
MOM: mitochondrial outer membrane
MOMP: mitochondrial outer membrane permeabilization
VDAC2: voltage-dependent anion channel 2
